# Metabolic Profiling of Hypoxic Cells Revealed a Catabolic Signature Required for Cell Survival

**DOI:** 10.1371/journal.pone.0024411

**Published:** 2011-09-02

**Authors:** Christian Frezza, Liang Zheng, Daniel A. Tennant, Dmitri B. Papkovsky, Barbara A. Hedley, Gabriela Kalna, David G. Watson, Eyal Gottlieb

**Affiliations:** 1 Cancer Research United Kingdom, The Beatson Institute for Cancer Research, Glasgow, United Kingdom; 2 Strathclyde Institute of Pharmacy and Biomedical Sciences, University of Strathclyde, Glasgow, United Kingdom; 3 Laboratory of Biophysics and Bioanalysis, Department of Biochemistry, University College Cork, Cork, Ireland; Laurentian University, Canada

## Abstract

Hypoxia is one of the features of poorly vascularised areas of solid tumours but cancer cells can survive in these areas despite the low oxygen tension. The adaptation to hypoxia requires both biochemical and genetic responses that culminate in a metabolic rearrangement to counter-balance the decrease in energy supply from mitochondrial respiration. The understanding of metabolic adaptations under hypoxia could reveal novel pathways that, if targeted, would lead to specific death of hypoxic regions. In this study, we developed biochemical and metabolomic analyses to assess the effects of hypoxia on cellular metabolism of HCT116 cancer cell line. We utilized an oxygen fluorescent probe in anaerobic cuvettes to study oxygen consumption rates under hypoxic conditions without the need to re-oxygenate the cells and demonstrated that hypoxic cells can maintain active, though diminished, oxidative phosphorylation even at 1% oxygen. These results were further supported by in situ microscopy analysis of mitochondrial NADH oxidation under hypoxia. We then used metabolomic methodologies, utilizing liquid chromatography–mass spectrometry (LC-MS), to determine the metabolic profile of hypoxic cells. This approach revealed the importance of synchronized and regulated catabolism as a mechanism of adaptation to bioenergetic stress. We then confirmed the presence of autophagy under hypoxic conditions and demonstrated that the inhibition of this catabolic process dramatically reduced the ATP levels in hypoxic cells and stimulated hypoxia-induced cell death. These results suggest that under hypoxia, autophagy is required to support ATP production, in addition to glycolysis, and that the inhibition of autophagy might be used to selectively target hypoxic regions of tumours, the most notoriously resistant areas of solid tumours.

## Introduction

The majority of solid cancers contain poorly oxygenated areas [1]. Cells react to oxygen limitation by genetic and biochemical reprogramming that decrease mitochondrial functions and drives glucose utilization for energy production [2]. The effects of oxygen availability on the balance between oxidative phosphorylation and glycolysis were observed more than a century ago by Louis Pasteur [3]. It is now acknowledged that part of the adaptation to hypoxia is mediated by numerous proteins among which the hypoxia inducible transcription factor HIF1 plays a major role [4]. When oxygen is limited HIF1α is stabilized and can bind to its partner HIF1β to form an active HIF1 complex which induces its target genes and leads to a complex metabolic rearrangement [5], [6], [7], [8], [9], [10], [11]. The effects of this rearrangement include the upregulation of glycolytic enzymes and the glucose transporter Glut1. In addition, HIF also regulates mitochondrial functions. It was recently shown that HIF1 stabilization under hypoxia leads to the expression of Pyruvate Dehydrogenase Kinase 1 (PDK1) [7], [10] a protein that phosphorylates and inactivates Pyruvate Dehydrogenase (PDH), limiting the conversion of pyruvate to Acetyl-CoA in the mitochondria. Consequently, PDK1 induction decreases the tricarboxylic acid (TCA) cycle activity and reduces oxygen consumption.

Hypoxia may thus affect energy metabolism both directly, by limiting the availability of oxygen to the mitochondria, and indirectly through transcription-mediated metabolic rearrangements. Therefore, it is crucial to develop reliable techniques for assessing mitochondrial functions under hypoxia. This is of particular importance when looking for general cellular metabolic changes imposed by low oxygen since the mitochondrion is a complex metabolic hub that not only provides energy for the cell but is also crucial for the generation of reducing equivalents (NADH and NADPH) and building blocks for anabolic reactions [12]. It is therefore reasonable to assume that hypoxic impairment of mitochondria may have metabolic effects beyond anaerobic glycolysis.

Metabolomics is a powerful tool developed to systematically analyse the metabolic fingerprint of a cell, widely used in the past to analyze microorganisms metabolism [13]. Metabolomic studies have shown that cellular metabolic networks are robust and the dynamic behaviour of biochemical pathways is governed by a highly interconnected regulatory system [14]. Our current challenge is to understand how this robust system is deregulated and perturbed under hypoxia and how this leads to a new metabolic fingerprint. In this work, the effect of hypoxia on cellular metabolism was analysed in HCT116 colorectal cancer cell lines using a combination of biochemistry, microscopy and metabolomics techniques. We demonstrated that these cells adapt to hypoxia by increasing anaerobic glycolysis and by decreasing respiratory rate and mitochondrial functions. The metabolomics approach further unveiled unexpected metabolic signature that demonstrated the importance of catabolic reactions for the survival of hypoxic cells.

## Results

### Assessment of the hypoxic phenotype

**Figure 1:**
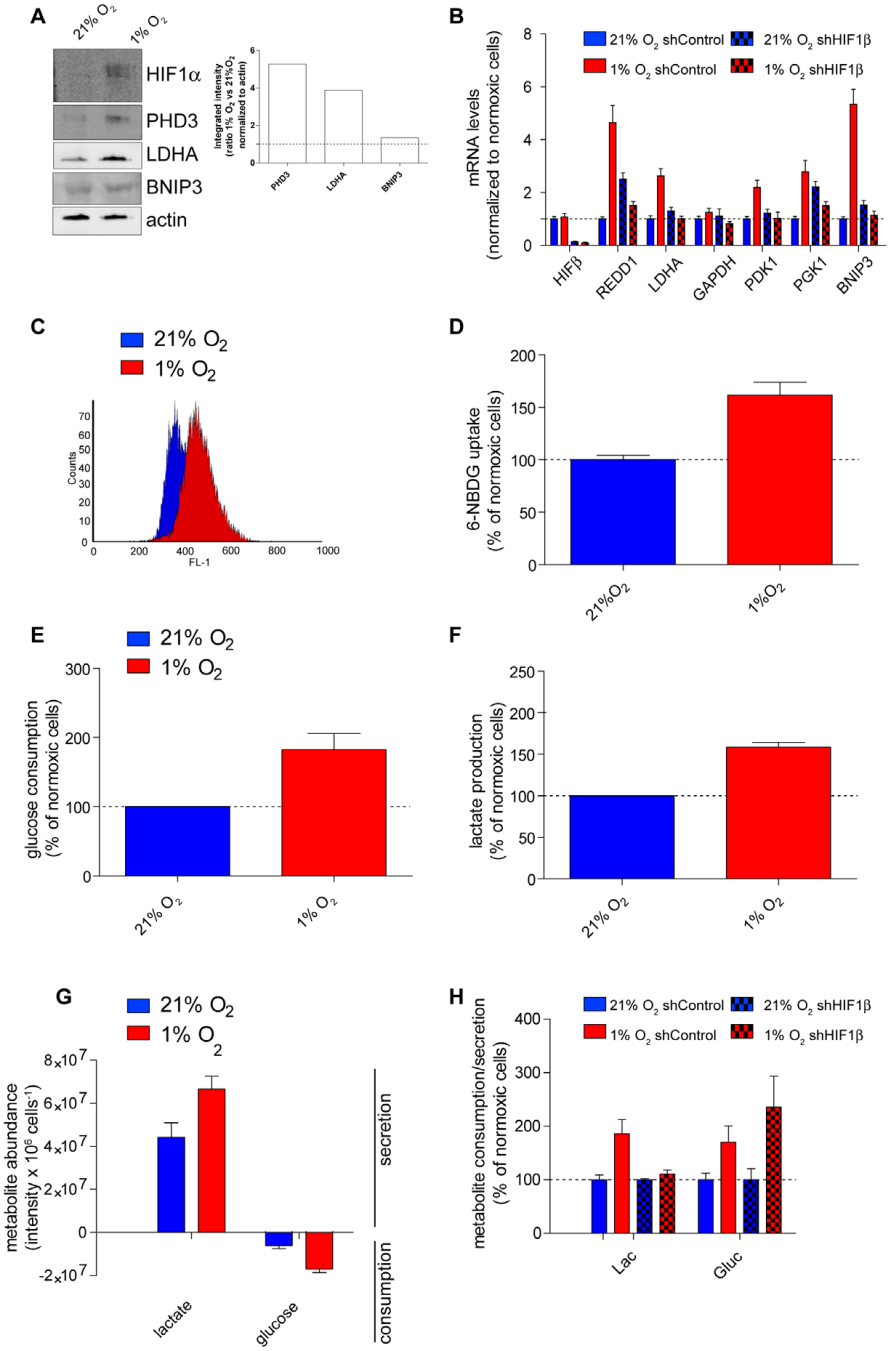
Biochemical signature of hypoxic cells. (A) HCT116 cells were exposed to normoxia (21% oxygen) or hypoxia (1% oxygen) for 36 hours and the protein levels of HIF1α and the indicated HIF targets were analysed by western blot. (B) HCT116 cells were transfected with either control short hairpin RNA (shControl) of shHIF1β and then incubated for 36 hours under normoxia or hypoxia. RNA was extracted and retrotranscribed to analyse the expression of HIF1β and the indicated HIF target genes by qRT-PCR. Where indicated, HIF transcriptional activity was inhibited by silencing the HIF transcriptional co-factor HIF1β (shHIF1β). (C–D) Cells were treated as in panel A and were then incubated with 30 µM of the fluorescent glucose analogue 6-NBDG for 10 minutes prior to analysis by fluorescence-activated cell sorter (FACS). (E–F) The levels of glucose (E) and lactate (F) in the medium were assessed enzymatically at 36 hours under normoxia or hypoxia. (G) LC-MS was utilized to measure glucose consumption and lactate production of HCT116 cells incubated for 36 hours under normoxia or hypoxia. (H) Cells were treated as in B and lactate excretion and glucose consumption was assessed by LC-MS analysis in the medium. Data are normalized to control-transfected cells under normoxia. Data are presented as means ± SE (n = 3). HIF1α/β, Hypoxia Inducible Factor 1α/β; PHD3, Prolyl Hydroxylase Domain proteins 3; LDHA, Lactate Dehydrogenase A; BNIP3, Bcl2 and E1B 19kD Interacting Protein; GAPDH, Glyceraldehyde 3-phosphate dehydrogenase; PDK1, Pyruvate Dehydrogenase Kinase 1; PGK1, Phosphoglycerate Kinase 1; REDD1, REgulated in Development and DNA damage response protein 1.

The cellular response to hypoxia was studied by culturing HCT116 (**ATCC® Number: CCL-247™**) cells either under normoxia (21% oxygen) or hypoxia (1% oxygen) for 36 hours. The chronic exposure to this oxygen tension recapitulates the oxygen levels found in hypoxic tumours [1] and has been shown to trigger a full hypoxic response in cell cultures. As a read-out of the genetic response to hypoxia in these experimental settings, HIF1α protein levels were analysed. HIF1α is a key transcription factor that, once stabilized under hypoxia, orchestrates many aspects of the metabolic adaptation to low oxygen level. As expected, chronic hypoxia led to HIF1α stabilization and to the induction of HIF1 target genes (Figure 1A). In particular, several glycolytic enzymes such as glyceraldehyde-3-phosphate dehydrogenase (GAPDH), phosphoglycerate kinase 1 (PGK1) and lactate dehydrogenase A (LDHA) were significantly upregulated in hypoxic cells (Figure 1B). Furthermore, the rate of glucose uptake, measured by the fluorescent derivative of glucose, 6-[N-(7-nitrobenz-2-oxa-1,3-diazol-4-yl)amino]-6-deoxyglucose (6NBDG), and the rate of glucose consumption, were both elevated in hypoxic cells (Figure 1C–G). In line with these observations, the increased glycolytic flux was mirrored by an increase in lactate production under hypoxia (Fig. 1F–G). Importantly, the relevant gene transcription pattern and lactate secretion in response to chronic hypoxia were inhibited when HIF1β was efficiently silenced in these cells, emphasising the role of HIF in long-term metabolic adaptation (Figure 1B and H). However, the increase in glucose uptake under hypoxia was not reversed by HIF silencing, indicating that bioenergetics needs can also be met by direct biochemical alterations without long-term genetic adaptation (Figure 1H).

### Mitochondrial function under hypoxia

**Figure 2:**
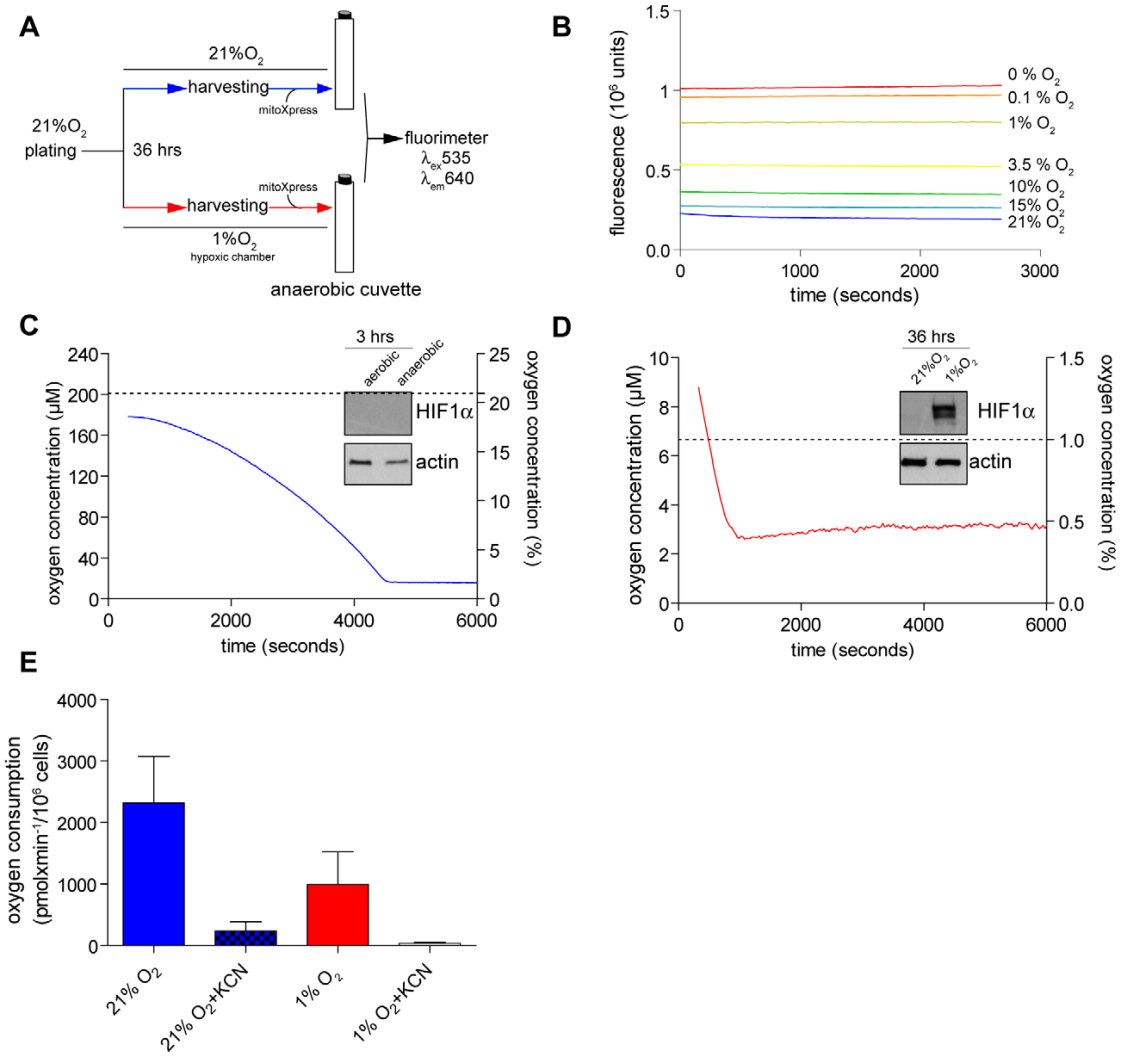
Measurement of respiration of hypoxic cells. (A) Schematic representation of the experiment for the assessment of respiration using an anaerobic cuvette and the oxygen sensor MitoXpress. (B) Fluorescence intensity calibration of MitoXpress at different oxygen tensions required for quantification of oxygen concentration. (C–D) Representative traces of oxygen consumption of equal amount of cells pre-incubated under normoxia (C) or hypoxia (D). Cells were analysed for their HIF1α content. Equal amounts of proteins were analysed by western blot and probed for HIF1α and actin (Inserts). (E) Quantification of oxygen consumption rates of normoxic and hypoxic cells. When indicated, cells were pre-incubated with 1 mM potassium cyanide for 5 minutes before starting the measurement of oxygen consumption. Data are presented as means ± SE (n = 3).

Mitochondria are crucial oxygen sensors, and their function is linked to several metabolic pathways. Therefore, mitochondrial function under normoxia and hypoxia was analysed. To overcome the technical limitations of standard polarography for measuring oxygen consumption of hypoxic cells, in particular, the requirement for samples reoxygenation, phosphorescence-quenching oxymetry was used. This method is compatible with standard fluorescent spectrometers and it retains sensitivity at low oxygen concentrations. Therefore, it enabled us for the first time to measure oxygen consumption of intact hypoxic cells without the need to reoxygenate the samples, as is commonly done by others (see Figure 2A for a schematic representation of the experiment). To convert the fluorescent signals into actual oxygen consumption rates, the probe was calibrated at different stable levels of dissolved oxygen (Figure 2B). Since the cells were maintained in sealed “anaerobic” cuvettes during the entire analysis, respiration rate could be measured across a range of oxygen levels from normoxia to anoxia. The cells showed a constant rate of oxygen consumption until deep hypoxia (estimated oxygen concentration below 1%) was reached in the anaerobic cuvette (Figure 2C). Of note, HIF1α was not yet stabilized when cells reached acutely low oxygen levels (Figure 2C; insert), suggesting that HIF does not play a role in the inhibition of mitochondrial respiration in response to acute hypoxia. Importantly, these results demonstrate that cells have the capacity to maintain constant respiration under a wide range of hypoxic conditions until oxygen becomes a limiting factor. We next measured the oxygen consumption rate of cells exposed to chronic hypoxia (36 hours at 1% oxygen). In line with the described above results, hypoxic cells retained mitochondrial-dependent oxygen consumption (Figure 2D) which was promptly inhibited by the cytochrome *c* oxidase inhibitor, potassium cyanide (KCN) (Figure 2E). However, respiratory rate of cells continuously grown at 1% oxygen was significantly below that of normoxic cells (Figure 2E).

**Figure 3:**
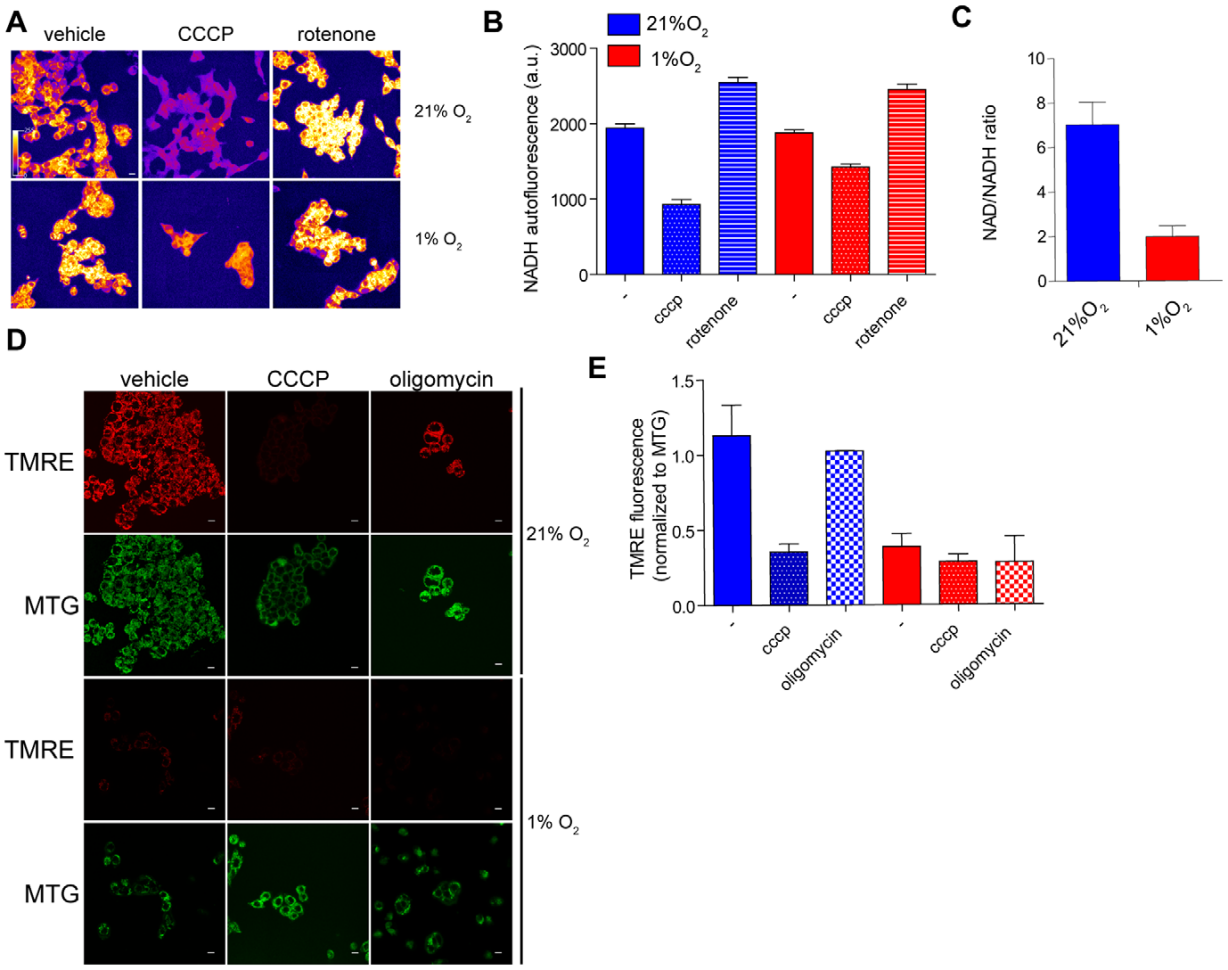
Analysis of NADH and mitochondrial membrane potential under hypoxia. (A–B) Representative images (A) and their quantifications (B) of NADH autofluorescence in normoxic and hypoxic cells left untreated or treated with the indicated drugs (10 µM CCCP or 10 µM Rotenone). The raw images were converted into “fire” LUT (shown in the top left image) using Image J. Bar  = 50 µm. (C) HCT116 cells were incubated under normoxia or hypoxia for 36 hours and whole cell extracts were assessed enzymatically for NAD^+^ and NADH levels. (D–E) Representative images (D) and their quantifications (E) of mitochondrial membrane potential of cells incubated under normoxia or hypoxia. Cells were stained with 10 nM TMRE and 50 nM MTG for 30 minutes. Cells were then left untreated or pre-incubated with 5 µM oligomycin for 30 minutes or 10 µM CCCP for 2 minutes. A whole cell z-stack was acquired and the maximum projection was used as representative images. Bar  = 50 µm.

Despite the fact that respiration is partially preserved under hypoxia, other parameters of mitochondrial function may be affected with important consequences on cellular metabolism. In particular, the decreased electron flux through the respiratory chain might cause a change in the ratio between the reduced (NADH) and oxidized (NAD^+^) nicotinamide adenine dinucleotide that may affect the cytosolic levels NAD^+^ required for glycolysis. To assess the levels of NADH in intact cells we took advantage of the spectral characteristic of the molecule, which is fluorescent when excited by ultra-violet light, while its oxidized form, NAD^+^, is not. Since NADH is the major autofluorescent signal present in cells, confocal real-time imaging of NADH has been widely used to detect changes in NADH oxidation and as readout of mitochondrial respiration [15], [16]. To properly evaluate the effect of mitochondrial respiration on NADH levels, cells were incubated with rotenone, an inhibitor of NADH-ubiquinone oxidoreductase (Complex I) and with the uncoupler carbonyl cyanide m-chlorophenylhydrazone (CCCP). In normoxic cells CCCP depleted mitochondrial NADH due to stimulation of the maximal rate of NADH-dependent respiration. On the other hand, rotenone, by blocking NADH oxidation, caused a maximal increase in mitochondrial autofluorescence (Figure 3A–B). Interestingly, hypoxic HCT116 cells showed minimal changes of basal NADH levels and rotenone could still increase NADH levels to that of rotenone-treated normoxic cells (Figure 3A–B). The response to CCCP of hypoxic cells was somewhat different than that of normoxic cells with only partial reduction in NADH levels (Figure 3A–B). This is in line with the reduced rate of respiration observed under hypoxia (Figure 2E) as NADH is the major electron donor to oxygen in the respiratory chain. These results underline the ongoing complex I-dependent NADH oxidation under hypoxia that would also explain the mitochondrial-dependent oxygen consumption under these conditions. In line with the above, a clear reduction in NAD^+^/NADH ratio was observed under hypoxic conditions indicating a more reduced state of the cells under hypoxia (Figure 3C).

The activity of the respiratory chain is mirrored by the generation of the mitochondrial membrane potential, the proton gradient across the inner mitochondrial membrane. Therefore, normoxic and hypoxic cells were incubated with the mitochondrial potential-dependent probe tetramethyl rhodamine ethyl ester (TMRE) together with the mitochondrial potential-independent probe Mitotracker green (MTG). Using confocal microscopy, cumulative images of sequential focal sections were acquired and z-stacks of the whole microscopic field were generated in order to measure the volume and intensity of the red and green markers. This ratiometric (red/green) quantification was required to reduce possible artefacts arising from changes in fluorescence emission of TMRE due to changes in mitochondrial shape and volume rather than from actual changes in mitochondrial membrane potential. Representative images were obtained by flattening the z-stack into a maximum projection image (Figure 3B). To rule out the possibility that mitochondrial membrane potential was generated by ATP hydrolysis and proton pumping by ATP synthase working in reverse, cells were also incubated with oligomycin, the inhibitor of the F_o_ subunit of ATP synthase. Our data showed that the normoxic cells incubated with oligomycin maintained their mitochondrial potential while, as expected, CCCP completely depolarized mitochondria (Figure 3B and D). Hypoxic cells showed a dramatic decrease in mitochondrial potential even in the absence of oligomycin (Figure 3B, bottom panels), suggesting that hypoxia triggers a decrease in mitochondrial potential that cannot be recovered by the reversal of ATP synthase. Subsequently, we analysed whether the mitochondrial dysfunction of hypoxic cells has a functional outcome on ATP levels due to the lack of mitochondrial membrane potential required for ATP synthesis. Indeed, hypoxic ATP levels dropped by about 40% compared to normoxic cells (Figure 3E). Importantly, a further drop in ATP levels was observed after a short incubation of hypoxic cells with oligomycin, suggesting that ATP levels of hypoxic cells are partially maintained by mitochondrial ATP synthase, despite the low mitochondrial potential.

### Metabolomic analysis of hypoxic cells

Oxygen limitation, mitochondrial dysfunction, and the genetic reprogramming initiated under hypoxia are likely to elicit global metabolic changes beyond anaerobic glycolysis. To better understand the metabolic signature of hypoxic cells, we performed an unsupervised endo-metabolomic analysis of normoxic and hypoxic cells using LC-MS. The total ion current of the different samples was used as a confirmation for normalized input (Figure S1A). The metabolomic profile of each experiment is a matrix of 241 annotated and confirmed metabolites (Table S1) in 6 columns (3 hypoxic and 3 normoxic samples). This matrix was subjected to multivariate statistical analysis. First, data was log(2) transformed in order to compress the dynamic range of peak intensities (Figure S1B–C). Importantly, the log(2) transformation did not affect the clustering of the samples. Principal component analysis (PCA), an unsupervised multivariate statistical method, was applied to the data matrix to identify significant clusters in an unsupervised manner. The PCA showed that the two experimental conditions are clearly statistically distinguishable (Figure S2A). This suggests that hypoxia caused an unambiguous change in the cell metabolome underlining the sensitivity of this unbiased metabolic tool to differentiate between metabolic states of cells. Due to possible fluctuation of metabolic status of the cells, a second independent metabolic experiment was performed and only metabolites that demonstrated consistent behaviour in both experiments were further evaluated.

**Figure 4:**
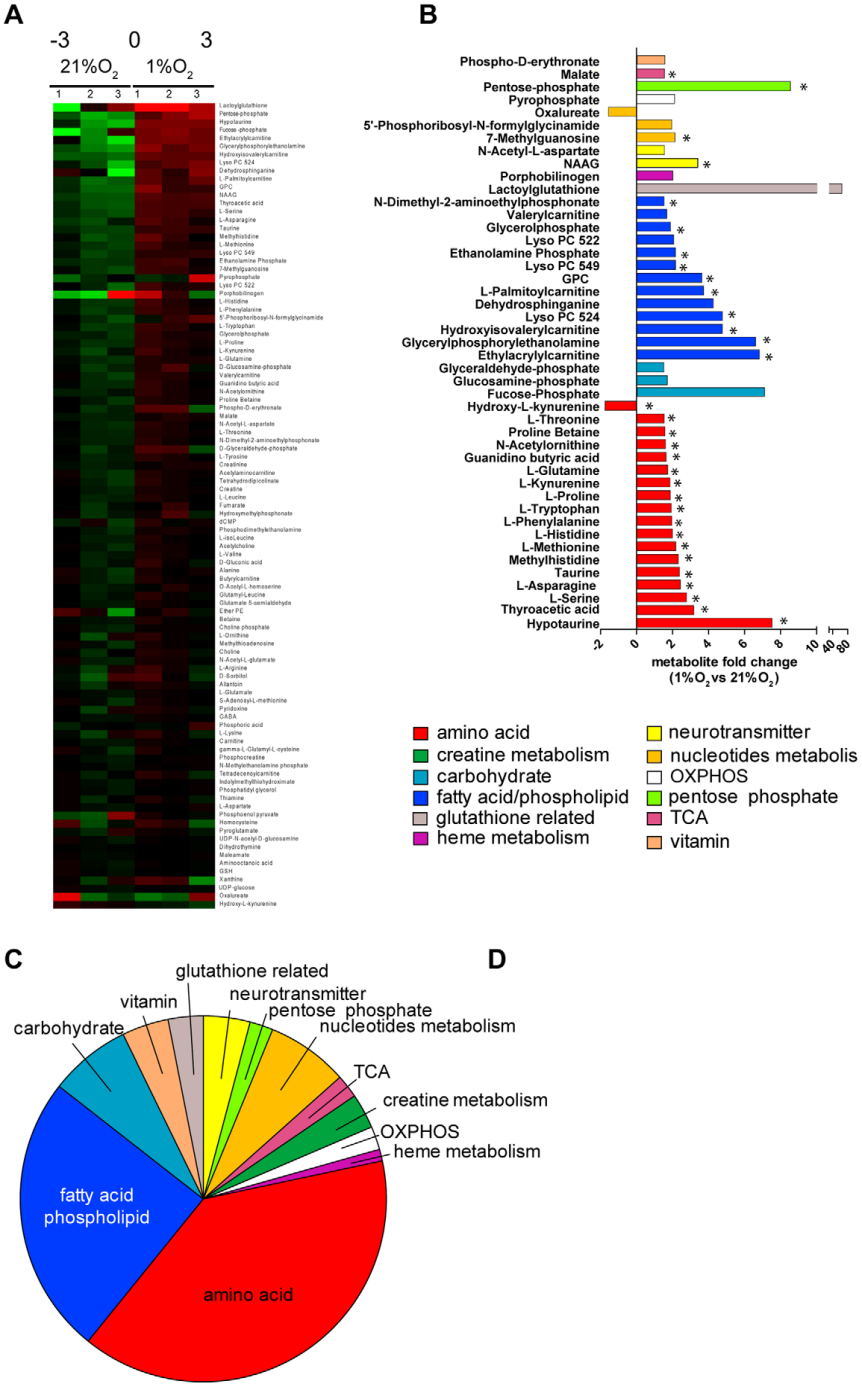
Metabolomic analysis of hypoxic cells. (A) Heatmaps of annotated metabolites with the same trend (upregulated or downregulated in hypoxia) in two independent experiments each one performed in triplicates. (B) Fold change variation of the selected metabolites (>1,5 fold). Significant changes (p<0.05) are indicated with an asterisk. Metabolites were assigned to metabolic pathways according to KEGG database. (C) Pie chart representation of the metabolic pathways which are significantly changed under hypoxia.

The unsupervised clustering analysis identified 99 annotated metabolites that are different between hypoxia and normoxia (Table S2). Among them, 45 metabolites showed consistent fold change of more than 1.5 (increase or decrease) in hypoxia and changes in 32 of them were statistically significant with a p-value below 0.05 (Figure 4A, B and Figure S2B). These compounds were clustered into different metabolic pathways as defined by the Kyoto Encyclopaedia of Genes and Genomes (KEGG) database. An enrichment of metabolites which are catabolic intermediates of proteins and lipids was clearly observed under hypoxia (Figure 4B, C and Table S2). These metabolites include kynurenine (tryptophan catabolite), and acyl-carnitine derivatives (fatty acid catabolites) to name a few. These results suggest that hypoxic HCT116 cells might rely on catabolic processes to compensate the energetic defect generated by the impairment of mitochondrial activity and that cannot be compensated by the increased glycolytic flux.

**Figure 5:**
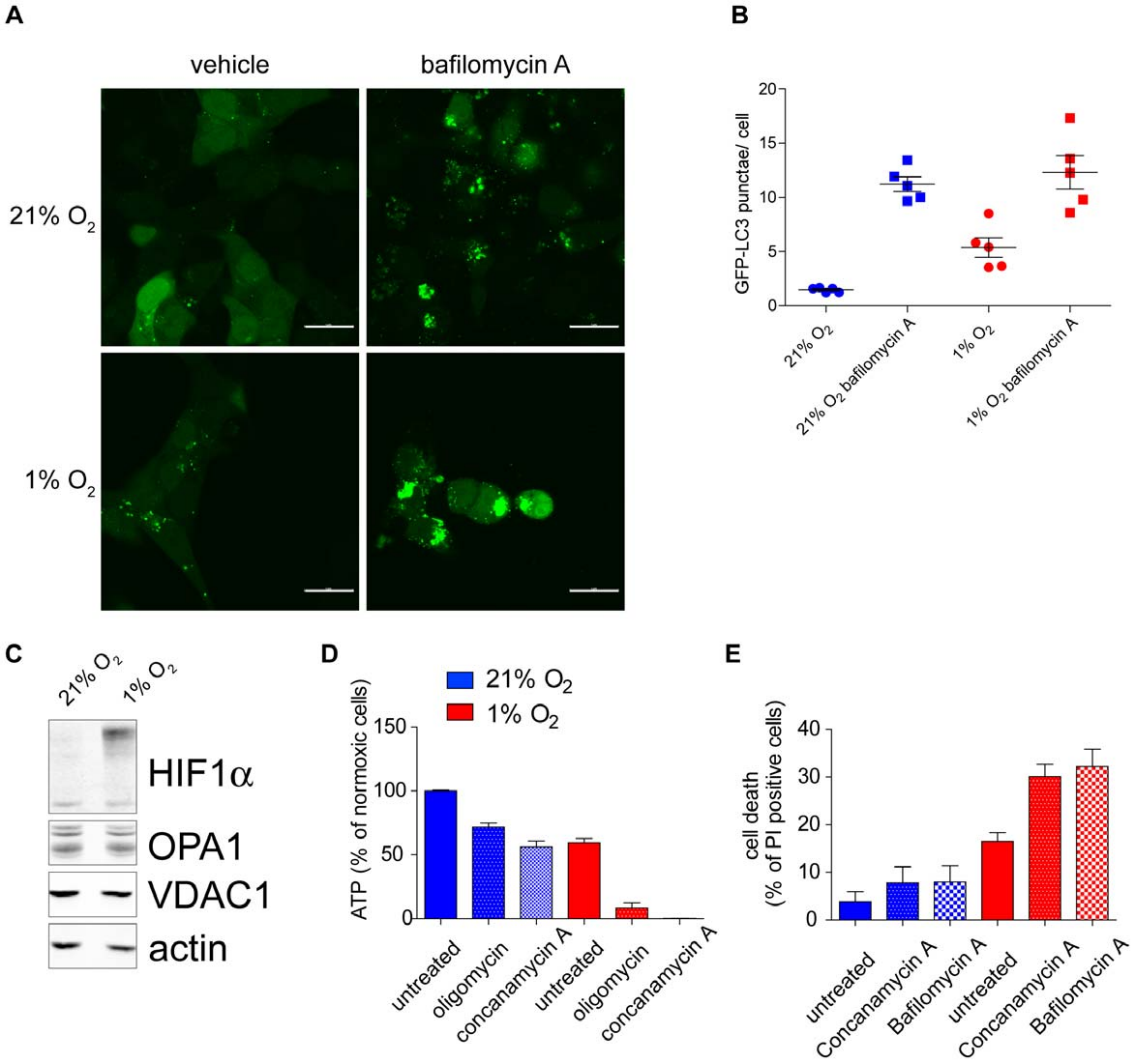
The role of autophagy in hypoxia. (A–B) representative images (A) and their quantifications (B) of cells stably expressing the autophagy reported GFP-LC3. As positive control cells were incubated with bafilomycin A, an inhibitor of the lysosomal functions which lead to the increased steady-state levels of autophagosomes. (C) The protein levels of HIF1α and the mitochondrial proteins OPA1 and VDAC1 in cells incubated under normoxia or hypoxia was assessed by western blot analysis. Actin was used as a loading control. (D) ATP levels were analysed in cells exposed to normoxia or hypoxia. Where indicated, cells were pre-incubated for 2 hours with 5 µM oligomycin (ATP synthase inhibitor) or 1 nM concanamycin A (autophagy inhibitor). Data are presented as means ± SE (n = 9). (E) Viability was measured in cells exposed to normoxia or hypoxia. Where indicated, cells were pre-incubated for 8 hours with 1 nM concanamycin A or 100 nM bafilomycin A. Cell viability was then assessed by propidium iodide exclusion by FACS. Data are presented as means ± SE (n = 9).

An important catabolic process which is known to occur under hypoxia is autophagy, the process of cell’s self-cannibalism that maintains nutrient supply under starvation [17], [18]. In order to validate the presence of autophagy in HCT116 cells under hypoxia we used the autophagic reported –GFP-LC3, which relocates from the cytosol to the autophagosomes during autophagy and hence appear more punctated when analysed microscopically [19]. Indeed, increased autophagosome formation could be clearly observed under hypoxia in these cells (Figure 5A, B). Importantly, BNIP3, a HIF-dependent gene that promote autophagy, was found to be significantly increased in hypoxic HCT116 cells (Figure 1A, B). Of note, a similar increase in BNIP3 mRNA levels was found in a transcriptomic study (microarray) of hypoxic HCT116 cells [10]. Since BNIP3 was associated with increased specific autophagy of the mitochondria (mitophagy) [20] we investigated whether some of the metabolic changes we observed under hypoxia were due to an active loss of the mitochondria via mitophagy. However, we could not identify changes in mitochondria levels in these cells when analysed either microscopically (Figure 2D) or by the levels of specific mitochondrial proteins (Figure 5C). Therefore, we concluded that while autophagy is induced in HCT116 cells after 36 hours under hypoxia, an accompanied mitochondria-specific elimination process was not detected and cannot account for the observed metabolic changes. To further corroborate the importance of this catabolic process as an integral part of the metabolic adaptation process to hypoxia, hypoxic cells were incubated with autophagy inhibitors, Concanamycin A or Bafilomycin A. Inhibition of autophagy resulted in a dramatic depletion of ATP levels in hypoxic HCT116 cells (Figure 5D) and in a significant loss of cell viability (Figure 5E).

## Discussion

The role of oxygen in the regulation of cellular metabolism has been widely investigated over more than a century. Initially, the cellular adaptation to hypoxia was mainly considered as a rearrangements of metabolite fluxes as a consequence of respiratory impairment [21]. The discovery of HIF and of other oxygen-dependent genes shifted the attention from biochemical to genetic adaptations to hypoxia. Interestingly, it now appears that biochemical and genetic changes observed under hypoxia are intertwined in complex regulatory pathways where metabolites regulate gene expression and gene expression regulates biochemical pathways therefore both contributing to the metabolic signature of hypoxic cells.

Mitochondria are the major oxygen consumers in the cell and are in the heart of several biochemical pathways involved both energy production and the generation of anabolic intermediates required for proliferation. Therefore, changes in mitochondrial function under hypoxia are likely to contribute to the altered metabolic profile of cells, making mitochondria important oxygen sensors. While the genetic reprogramming during hypoxia has been characterized and many players in this process have been identified, the contribution of mitochondria to the hypoxic response is still uncertain. Isolated mitochondria can respire efficiently even at very low oxygen concentration [22] with a K_m_ for oxygen of less than 1 µM (∼0.1% oxygen saturation), advocating that mitochondrial metabolism is insensitive to changes in the physiological range of oxygen tensions. However, biochemical changes such as increased lactate production, alterations in the redox state of the cells and in ATP/ADP ratio were demonstrated at oxygen levels at which respiration was unaffected (i.e. >100 µM) [23], [24]. In a possible unifying scenario, it was proposed that in order to keep mitochondrial respiration constant until very low oxygen concentration is reached, other parameters of mitochondrial function, such as NADH oxidation and ATP production, must constantly be adjusted, thus affecting other pathways in the cytosol. While NADH and ATP availability are known to affects glycolytic flux, the effects of oxygen limitation on cellular metabolism may be wider than simply the induction of anaerobic glycolysis. In this investigation, we provide a novel experimental approach to assess the hypoxic signature of cancer cells by using a combination of biochemistry, real-time imaging and metabolomics to investigate several unanswered questions about metabolism of hypoxic cells.

Using phosphorescent-quenching oxymetry, respiration of normoxic and hypoxic cells was characterised. This technique allows for the measurement of oxygen consumption of intact hypoxic cells without the need to reoxygenate the sample. The sharp transition from an active respiring stage to a non-respiring stage suggests that cells reach near anoxia before respiration halts, confirming the capacity of mitochondria to respire at very low oxygen levels (Figure 2C). Importantly, the rate of respiration of cells adapted to chronic hypoxia demonstrated that they retain active respiration though at significantly reduced rate (Figure 2D and E). These results indicate that in chronically-hypoxic cells, oxygen consumption is limited even at 1% (∼10 µM) oxygen, concentration which is 10 folds above the reported K_m_ of mitochondria for oxygen. Changes in respiration at this oxygen levels might be explained either by changes in the V_max_ of cytochrome *c* oxidase [25] or by the effects of HIF on the composition of the respiratory chain subunits or on substrates availability to mitochondria [5], [10]. In addition, the decrease in respiration under hypoxia was linked to increased mitophagy [20]; however, confocal microscopy did not reveal gross changes in the mitochondrial content under hypoxia (Figure 3).

To overcome a bioenergetic deficit, metabolic alterations may be exploited by the hypoxic cell. Indeed, metabolomic analysis was demonstrated to be an effective tool to characterise the metabolic signature of hypoxic cells. PCA showed that distinct metabolic signatures of normoxic and hypoxic cells could be defined. The top-rank compounds (p-value <0.05 and fold change >1.5) found in hypoxic cell line showed extensive alterations of amino acids and fatty acid/phospholipids metabolism (Table S2). Among these, we observed an increase in the amino acids threonine, proline and glutamine. An increase in intracellular amino acids under hypoxia may be attributed to several possibilities: An increase in amino acid uptake and/or a decrease in protein synthesis, a phenomenon which is considered to be part of the metabolic adaptation to hypoxia where ATP-consuming reactions, like protein synthesis, are dramatically decreased [26]. Another possibility, which is not mutually exclusive, is that during hypoxia cells undergo protein catabolism in order to provide the cells with metabolic support and the increase in macroautophagy under hypoxia may provide an explanation to this observation [27]. In support of this, degradation products of amino acids were a significant part of the hypoxic signature (Table S2) and increase autophagy was observed under hypoxic conditions (Figure 5A, B). Fatty acid and phospholipid metabolic pathways are a second important metabolic signature of hypoxic cells (Figure 4). A significant increase in acyl-carnitines, such as butyl and valeryl carnitine, was identified in these cells. It is known that fatty acid oxidation for energy purposes accounts for a significant proportion of oxygen consumption in cells [28]. Therefore, the catabolic process of amino acids and fatty acid may be required to sustain the observed oxidative phosphorylation in hypoxic cells – freeing more glucose to be converted to lactate (and with it the recycling of cytosolic NAD^+^) and hence supporting faster glycolytic rate. In line with that, a dramatic reduction in ATP was observed in hypoxic cells under conditions where autophagy was inhibited (Figure 5D). Interestingly, acyl-carnitines themselves facilitate the autophagic process [29], [30].

This hypoxic metabolic profiling revealed a shift in balance between anabolic and catabolic processes that is likely to have important survival consequences. Therefore, unbalancing the anabolic/catabolic ratio under hypoxia, by either stimulating anabolism (e.g. by inhibiting AMPK) and/or inhibiting catabolism (e.g. blocking macroautophagy) is a possible strategy for specifically targeting hypoxic cells in tumours. In support to this hypothesis, the incubation with inhibitors of macroauthophagy such as concanamycin A and bafilomycin A caused significant cell death of hypoxic cells (Figure 4D). Taken together, our results underline the importance of combining biochemistry and metabolomics analyses to identify adaptation to stress induced by hypoxia. Importantly, the finding of specific adaptive pathways that operate under hypoxia might offer new therapeutic approaches for cancer treatment by targeting the metabolic pathways required for the survival of hypoxic tumour cells and hence sparing normal cells, which are rarely exposed to hypoxia.

## Materials and Methods

Detailed protocols are available on Supporting Information online in the file [Supplementary-material pone.0024411.s005].

## Supporting Information

Figure S1 **Metabolomic analysis of hypoxic cells.** (A) Total ion current of cell extracts obtained from hypoxic or normoxic HCT116 submitted to LC-MS analysis. (B–C) Histograms of the distribution of m/z of metabolites before (B) and after (C) log2 transformation obtained from LC-MS analysis.
(TIF)

Figure S2 **Metabolic signature hypoxic cells.** (A) Spectral clustering of cells under normoxia or hypoxia. (B) Heatmaps of annotated metabolites with the same trend (upregulated or downregulated in hypoxia) using the indicated restrictions for fold change (f.c.) and p-value.
(TIF)

Table S1 **Metabolomic database.** Database of annotated compounds detectable by Orbitrap Exactive LC-MS by negative and positive ion mode. RT =  retention time.
(XLS)

Table S2 **Metabolic signature of hypoxic cells.** List of metabolites changed upon hypoxia. Metabolites with a cut off of fold change of >1.5 or p-value <0.05 are indicated with an asterisk. The metabolic pathways (derived from KEGG) which each metabolite belongs to are indicated. Metabolic pathway coding are as follow: A: amino acid, C: carbohydrate, E: creatine, F: fatty acid/phospholipids, G: glutathione, H: heme, N: neurotransmitter, Nu: nucleotide, O: oxidative phosphorylation, P: pentose phosphate, T: TCA cycle, V: vitamins.
(XLS)

Methods S1 **Detailed description of the materials and methods utilized in the manuscript.**
(DOCX)

## References

[1] Hockel M, Vaupel P (2001). Tumor hypoxia: definitions and current clinical, biologic, and molecular aspects.. J Natl Cancer Inst.

[2] Semenza GL (2010). Oxygen homeostasis.. Wiley Interdiscip Rev Syst Biol Med.

[3] Krebs HA (1972). The Pasteur effect and the relations between respiration and fermentation.. Essays Biochem.

[4] Semenza GL (1998). Hypoxia-inducible factor 1: master regulator of O2 homeostasis.. Curr Opin Genet Dev.

[5] Fukuda R, Zhang H, Kim JW, Shimoda L, Dang CV (2007). HIF-1 regulates cytochrome oxidase subunits to optimize efficiency of respiration in hypoxic cells.. Cell.

[6] Hervouet E, Cizkova A, Demont J, Vojtiskova A, Pecina P (2008). HIF and reactive oxygen species regulate oxidative phosphorylation in cancer.. Carcinogenesis.

[7] Kim JW, Tchernyshyov I, Semenza GL, Dang CV (2006). HIF-1-mediated expression of pyruvate dehydrogenase kinase: a metabolic switch required for cellular adaptation to hypoxia.. Cell Metab.

[8] Luo W, Hu H, Chang R, Zhong J, Knabel M (2011). Pyruvate Kinase M2 Is a PHD3-Stimulated Coactivator for Hypoxia-Inducible Factor 1.. Cell.

[9] O'Hagan KA, Cocchiglia S, Zhdanov AV, Tambuwala MM, Cummins EP (2009). PGC-1alpha is coupled to HIF-1alpha-dependent gene expression by increasing mitochondrial oxygen consumption in skeletal muscle cells.. Proc Natl Acad Sci U S A.

[10] Papandreou I, Cairns RA, Fontana L, Lim AL, Denko NC (2006). HIF-1 mediates adaptation to hypoxia by actively downregulating mitochondrial oxygen consumption.. Cell Metab.

[11] Puissegur MP, Mazure NM, Bertero T, Pradelli L, Grosso S (2011). miR-210 is overexpressed in late stages of lung cancer and mediates mitochondrial alterations associated with modulation of HIF-1 activity.. Cell Death Differ.

[12] Frezza C, Gottlieb E (2009). Mitochondria in cancer: not just innocent bystanders.. Semin Cancer Biol.

[13] Reaves ML, Rabinowitz JD (2011). Metabolomics in systems microbiology.. Curr Opin Biotechnol.

[14] Grimbs S, Selbig J, Bulik S, Holzhutter HG, Steuer R (2007). The stability and robustness of metabolic states: identifying stabilizing sites in metabolic networks.. Mol Syst Biol.

[15] Mayevsky A, Rogatsky GG (2007). Mitochondrial function in vivo evaluated by NADH fluorescence: from animal models to human studies.. Am J Physiol Cell Physiol.

[16] Mayevsky A (1984). Brain NADH redox state monitored in vivo by fiber optic surface fluorometry.. Brain Res.

[17] Kroemer G, Marino G, Levine B (2010). Autophagy and the integrated stress response.. Mol Cell.

[18] Wilkinson S, O'Prey J, Fricker M, Ryan KM (2009). Hypoxia-selective macroautophagy and cell survival signaled by autocrine PDGFR activity.. Genes Dev.

[19] Kabeya Y, Mizushima N, Ueno T, Yamamoto A, Kirisako T (2000). LC3, a mammalian homologue of yeast Apg8p, is localized in autophagosome membranes after processing.. EMBO J.

[20] Zhang H, Bosch-Marce M, Shimoda LA, Tan YS, Baek JH (2008). Mitochondrial autophagy is an HIF-1-dependent adaptive metabolic response to hypoxia.. J Biol Chem.

[21] Henderson AR (1969). Biochemistry of hypoxia: current concepts. I. An introduction to biochemical pathways and their control.. Br J Anaesth.

[22] Chance B (1965). Reaction of oxygen with the respiratory chain in cells and tissues.. J Gen Physiol.

[23] Wilson DF, Erecinska M, Drown C, Silver IA (1977). Effect of oxygen tension on cellular energetics.. Am J Physiol.

[24] Wilson DF, Erecinska M (1985). Effect of oxygen concentration on cellular metabolism.. Chest.

[25] Chandel NS, Budinger GR, Choe SH, Schumacker PT (1997). Cellular respiration during hypoxia. Role of cytochrome oxidase as the oxygen sensor in hepatocytes.. J Biol Chem.

[26] Hochachka PW, Buck LT, Doll CJ, Land SC (1996). Unifying theory of hypoxia tolerance: molecular/metabolic defense and rescue mechanisms for surviving oxygen lack.. Proc Natl Acad Sci U S A.

[27] Papandreou I, Lim AL, Laderoute K, Denko NC (2008). Hypoxia signals autophagy in tumor cells via AMPK activity, independent of HIF-1, BNIP3, and BNIP3L.. Cell Death Differ.

[28] Whitmer JT, Idell-Wenger JA, Rovetto MJ, Neely JR (1978). Control of fatty acid metabolism in ischemic and hypoxic hearts.. J Biol Chem.

[29] Watanabe H, Kobayashi A, Hayashi H, Yamazaki N (1989). Effects of long-chain acyl carnitine on membrane fluidity of human erythrocytes.. Biochim Biophys Acta.

[30] Piper MH, Sezer O, Schwartz P, Hutter JF, Schweickhardt C (1984). Acyl-carnitine effects on isolated cardiac mitochondria and erythrocytes.. Basic Res Cardiol.

