# Supplementary figures and images for: Metabolic Profiling of Hypoxic Cells Revealed a Catabolic Signature Required for Cell Survival
Source: PLoS One. 2011 Sep 2;6(9):e24411. doi: 10.1371/journal.pone.0024411 (PMC3166325; doi:10.1371/journal.pone.0024411)

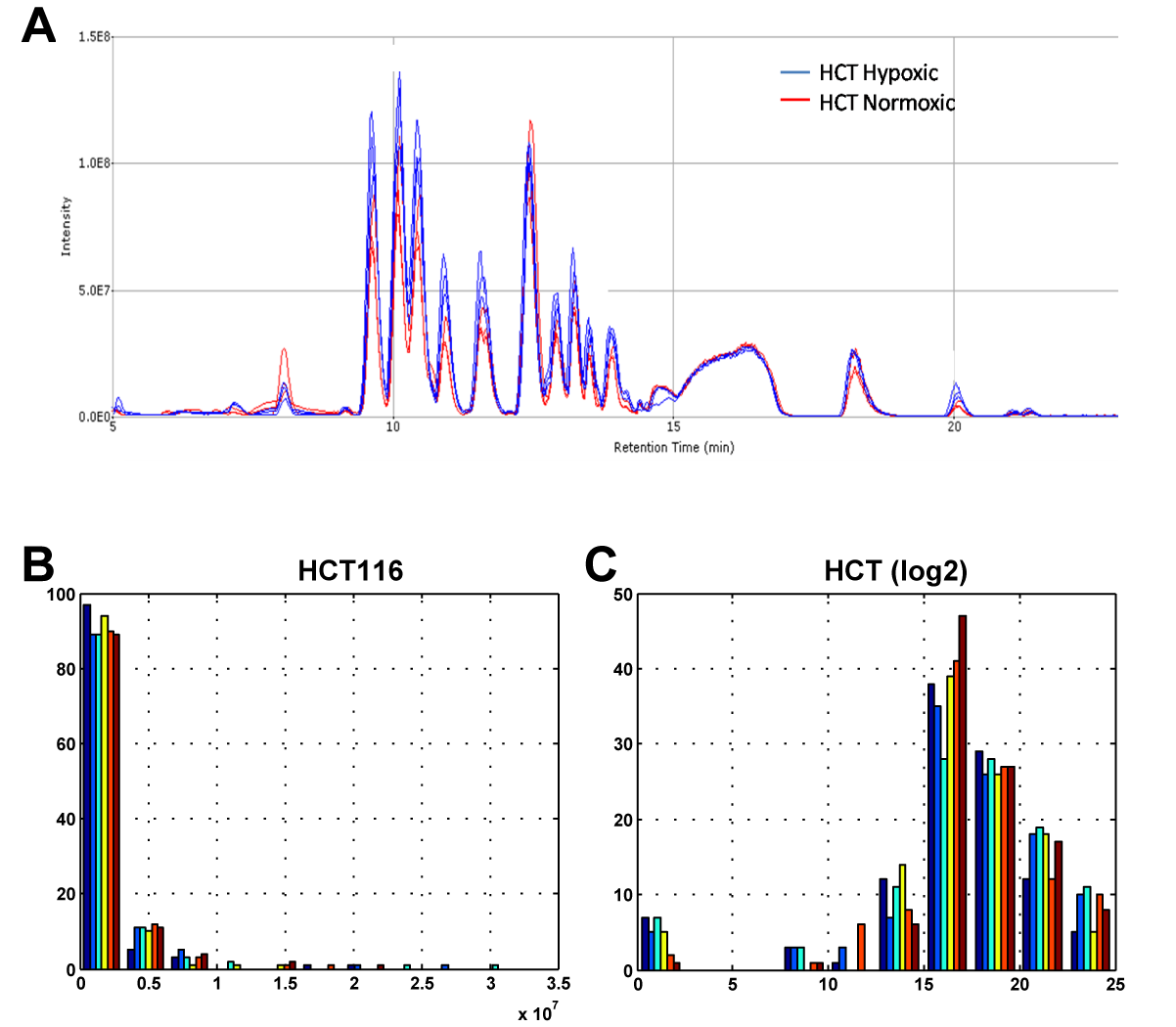

Supplement: Figure S1 — Metabolomic analysis of hypoxic cells. (A) Total ion current of cell extracts obtained from hypoxic or normoxic HCT116 submitted to LC-MS analysis. (B–C) Histograms of the distribution of m/z of metabolites before (B) and after (C) log2 transformation obtained from LC-MS analysis. (TIF) [file pone.0024411.s001.tif]

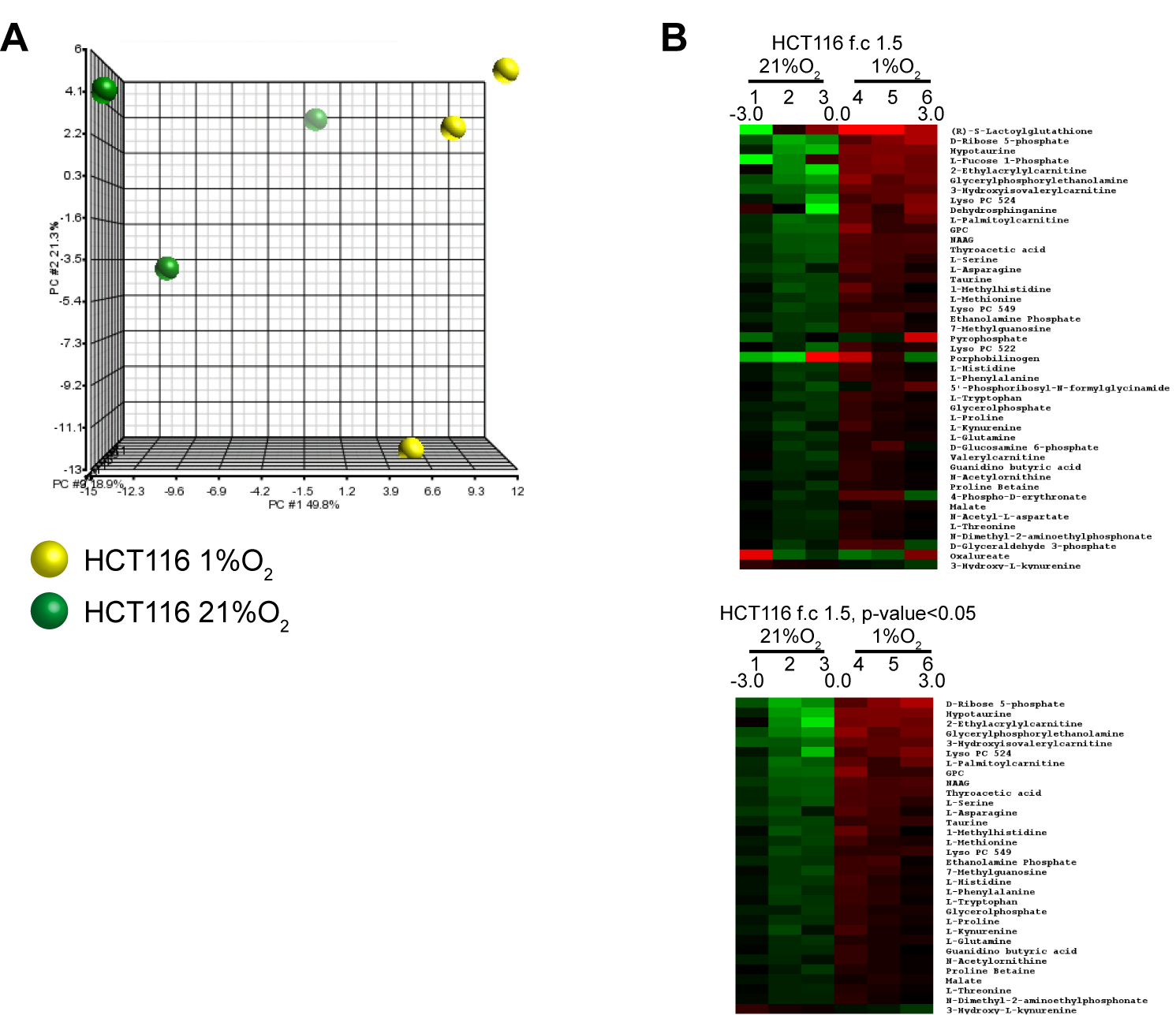

Supplement: Figure S2 — Metabolic signature hypoxic cells. (A) Spectral clustering of cells under normoxia or hypoxia. (B) Heatmaps of annotated metabolites with the same trend (upregulated or downregulated in hypoxia) using the indicated restrictions for fold change (f.c.) and p-value. (TIF) [file pone.0024411.s002.tif]
