# Supplementary material for: Metabolic Profiling of Hypoxic Cells Revealed a Catabolic Signature Required for Cell Survival
Source: PLoS One. 2011 Sep 2;6(9):e24411. doi: 10.1371/journal.pone.0024411 (PMC3166325; doi:10.1371/journal.pone.0024411)
Supplement: Methods S1 — Detailed description of the materials and methods utilized in the manuscript. (DOCX) [file pone.0024411.s005.docx]

**Methods S1**

**Cell cultures**

Colorectal adenocarcinoma cell line HCT116 purchased from ATCC® (Number CCL-247™) were cultured using standard procedures in a 37 °C humidified incubator with 5% CO_2_ in high-glucose Dulbecco's modified Eagle's medium (Invitrogen) supplemented with 10% heat-inactivated fetal bovine serum and 2 mM glutamine. For the hypoxic experiments, cells were plated under normoxic conditions and then moved in a humidified Invivo2 500 hypoxic chamber (Biotrace, Bridgend, UK) controlled by a Ruskin hypoxic gas mixer at 37 °C with 1% O_2_, 5% CO_2_ and 94% N_2_. HCT116 cells stably expressing GFP-LC3 were generated by retroviral infection of parental HCT116 cells with pBabe Puro-GFP-LC3. Infected cells were then selected using 1 µg/ml puromycin and a single population of GFP positive cells was isolated and expanded.

**Metabolomic analysis**

7x10^5^ cells were incubated under 21% or 1% O_2_ for 36 hours and then quickly washed with ice cold PBS on an ice bath. Cells were then lysed with a dry ice/methanol solution (-80 °C) of 80% methanol in water and quickly scraped. The insoluble material was immediately pelleted in a cooled centrifuge (0 °C) and the supernatant was collected for subsequent LC-MS analysis. Extraction of hypoxic cells was performed in the hypoxic chamber. The amount of extraction solution was calculated according to the number of cells present in the sample dish, extrapolated using a “counter dish” cultured in the same conditions of the sample dishes. A concentration of 1ml per 2x10^6^ cells was used in the extraction solutions.

**Cellular respiration using mitoXpress**

7x10^5^ cells were plated into 15 cm dishes and growth as indicated. Cells were then gently scraped, counted using a cell counter (CASY 1; Schärfe Systems, Reutlingen, Germany), and put into an anaerobic quartz cuvette (Starna Scientific, Essex, UK), with 50 μL of 100 μM MitoXpress solution. The cuvette was then sealed and moved into the fluorimeter’s lodge. The counting of hypoxic cells was extrapolated from a “counter dish” obtained by plating and growing cells in the same conditions as the sample dishes but used only for counting. For the measurement of hypoxic samples all the materials, media and probe were equilibrated under hypoxic conditions for at least 24 hours. The phosphorescence of the probe was followed in real time using 535 nm for excitation and 640 nm for emission, slit 10 nm, using a temperature-controlled fluorimeter set at 37 °C and a magnetic stirrer (Fluoromax 3, Jobin Yvon Horiba, Stanmore, UK), acquisition and integration time 30 seconds. When indicated, 1mmol/L potassium cyanide was added to the cell suspension prior the sealing of the cuvette.

**Glucose consumption, Lactate production and ATP levels**

2x10^5^ cells were grown on 6 well plates as indicated and the culture medium was used directly in the specified assays as per manufacturer's instructions. Lactate levels were assayed spectrophotometrically at 540 nm using the Lactate Kit (Trinity Biotech, Theale, UK). Glucose concentration was assayed spectrophotometrically at 540 nm using the Glucose Assay Kit (Sigma). For measurement of ATP, cells were washed twice with PBS in order to remove dead cells, lysed and ATP was measured according to manufacturer’s instructions.. All the previous parameters were normalized for total protein contents of the cell lysate measured by BCA assay (Thermoscientific) using BSA as standard.

Glucose uptake was measured by incubating cells with 30 μM glucose analogue 6-NBDG (Invitrogen, Paisley, UK) for 10 minutes. Cells where then washed, trypsinized and their fluorescence (λex: 465 nm, λem: 540nm) was measured by flow cytometry (FACSCalibur; Beckton Dickinson, Oxford, UK).

**Immunoblotting**

2x10^5^ cells were grown in the indicated conditions and then were extracted in Laemmli Sample buffer. The extraction of hypoxic cells was carried out in hypoxic conditions using pre-equilibrated reagents. The extraction of cells kept in the anaerobic cuvette (either open or closed screw cap) was performed by resuspending equal amounts of cells in Laemmli buffer, under hypoxic conditions. Equal amounts of protein were loaded into 4-12% Tris-Mops pre-casted gels (Invitrogen) and electrophoretically separated. After electrophoresis, the proteins were blotted onto 0.22 µm nitrocellulose (Millipore) and probed for the following antibodies: HIF1α (BD Biosciences, 610958), actin (AC-40; Sigma, Gillingham, UK), BNIP3 (Sigma,B7931), LDHA (Abcam, Ab92903), PHD3 (Abcam, ab 4562), OPA1 (BD Biosciences, 612602), VDAC (Sigma, V2139). Secondary antibodies were purchased from Licor: anti mouse 800 (962-32212), anti rabbit (926-32213), anti goat (926-32214). Integrated intensity was measured using the dedicated function of the Odissey software and normalized to actin.

**qRT-PCR analysis**

2x10^5^ cells were grown as indicated and mRNA was isolated as per manufacturer’s instructions using QIAshredder and RNEasy kits (Qiagen). mRNA was retro-transcribed into cDNA using random hexamers and MuMLV reverse transcriptase (Finnzymes) and qPCR reaction was performed on ABI 7500 Fast Thermocycler using GADPH and PGK primers designed as follow: GAPDH: 5’:agccaccatcgctcagacac 3’: gcccaatacgaccaaatcc; PGK1: 5’: ggagaacctccgctttcat 3’: gctggctcggctttaacc; BNIP3: 5’ gaatttctgaaagttttccttcca, 3’ ttgtcagcgccttccaat; PDH3: 5’ atcgacaggctggtcctcta, 3’ gatagcaagccaaccattgc; LDHA: 5’ tctctgtagcagatttggcaga, 3’ aagacatcatcctttattccgtaaa, REDD1: 5 ctggacagcagcaacagtg, 3’ acaccccatccaggtaagc; HIFβ (ARNT): 5’-ctacccgctcaggcttttc, 3’ caccaaactgggaagtacgag. qRT-PCR was performed in triplicate and normalized to nuclear lamin A designed as follow: 5’: tccctaaacagcaagccatc 3’: cacagaattgtgaagacaattacatc. Expression levels of the indicated genes were calculated by the ΔΔC_t_ method using the dedicated ABI software.

**Stable shRNA infection of HIF1β (ARNT)**

2x10^5^ cells were infected with shRNA for HIF1β and control plasmid using lentiviral infection. HEK293T and relevant packaging plasmids were used to produce the lentiviral suspension. shRNA plasmids (pLKO backbone) were purchased from Openbiosystems and the clone number TRCN0000003819 was used for subsequent infections. After infection, cells were selected using 4 mg/mL puromycin.

**NADH ratio measurement**

2 x 10^5^ were pelletted in a micro-centrifuge tube (2000 rpm for 5 min). Cells were then extracted with 400 µl of NADH/NAD Extraction Buffer ( 20 mM Sodium Bicarbonate, 100 mM Sodium Carbonate, 10 mM nicotinamide, 0.05% Triton X-100, pH= 10.3) by freeze/thaw two cycles (20 min on dry-ice, then 10 min at room temperature). The suspension was then mixed for 10 seconds and centrifuged at 14000 g for 5 min. 200 µl of the extracted samples were heated to 60 °C for 30 minutes in a heating block to decompose NAD^+^. NADH was measured using a cycling reaction occurring between MTT and alcohol dehydrogenase. The cycling buffer contains 100 mM TRIS-HCl, 5 mM EDTA, 20 mM MTT, 0.2 mg/mL alcohol dehydrogenase and 20 mM PES. The reaction was started by the addition of 2% EtOH. The absorbance was followed at 570 nM.

NAD/NADH Ratio is calculated as: [NADt – NADH]/NADH

**NADH autofluorescence measurements**

2x10^5^ cells were grown in 3.5 cm glass-bottom dishes (MatTek coprporatio, Ashland, USA) under normoxia or hypoxia and treated as indicated. The final concentrations of the drugs are: rotenone 5 μmol/L, carbonylcyanide-m-chlorophenyl hydrazone (CCCP) 10 μmol/L. Images were collected with a Leica TCS SP2 AOBS inverted laser scanning microscope equipped with a Coherent 351-364 nm UV laser using a 63x/1.32 ph3 oil HCX PL APO objective, resolution 1024X1024 dpi, 8-bit image depth, 200 Hz scanning speed, and line average 2. Laser excitation was 364 nm for NADH autofluorescence while fluorescence emission was adjusted with the AOBS at 390-486 nm. To increase sensitivity of NADH detection the pinhole aperture was 2.62 airy units. Each experiment was performed on a random field and a z-stack of the whole cell volume (1μm depth) was then acquired. For visual representation of NADH fluorescence, the maximum projection image was converted into a “fire” look up table (LUT) using the software Image J. The maximum projection of the z-stack was used to quantify NADH fluorescence using the Multimeasure plug-in of ImageJ after background subtraction using 150 pixel rolling ball radius. To measure NADH of hypoxic cells, dishes grown under hypoxia were sealed with parafilm and quickly visualized to avoid reoxygenation.

**Mitochondrial Potential measurements**

2x10^5^ cells were grown in 3.5 cm glass-bottom dishes (MatTek coprporation, Ashland, USA) under normoxia or hypoxia. Where indicated cells were loaded with 50 nmol/L MTG (Invitrogen) and 10 nmol/L Tetramethyl Rhodamine Ethyl Ester (TMRE) (Invitrogen) for 30 minutes and then left untreated or treated as indicated. The final concentrations of the drugs are: oligomycin 5μmol/l, rotenone 5 μmol/L, carbonylcyanide-m-chlorophenyl hydrazone (CCCP) 10 μmol/L. Cells were imaged using the Nikon A1R confocal microscope built on a Eclipse Ti-E inverted microscope equipped with a laser system with AOTF at 405nm and 561 nm using a 60x 1.3 NA Plan apochromat objective. A whole cell z-stack (0.2 μm depth) was acquired using the resonant scanning mode (30 fps) at 512 x 512 resolution, line average 4, 1 airy unit using a Piezo Z-stack motorized stage. A maximum projection of the z-stack has been used for representative images. To measure mitochondrial potential of hypoxic cells, dishes grown under hypoxia were sealed with parafilm and quickly visualized to avoid reoxygenation. A volumetric reconstruction was then obtained using the software Imaris and the MTG volume was used as a reference volume over imposed to the red channel for calculation of the TMRE volume’s intensity. A ratio between the two intensities was then provided as a read-out of mitochondrial potential.

**LC3 measurement**

2x10^5^ HCT116 cells stably expressing LC3-GFP were grown in 3.5 cm glass-bottom dishes (MatTek corporation, Ashland, USA) under normoxia or hypoxia. Cells were imaged using the Nikon A1R confocal microscope built on a Eclipse Ti-E inverted microscope equipped with a laser system with AOTF at 405nm and 561 nm using a 60x 1.3 NA Plan apochromat objective. LC3-GFP punctae and total cell number per field were then counted and punta/cell index was calculated. Cells incubated with 200 nM bafilomycin for 8 hours either under normoxia or hypoxia were used as positive control.

**Cell death assessment**

2x10^5^ cells were grown on 6 well plates under normoxia or hypoxia for 36 hours. 8 hours before harvesting cell were incubated with 100 nmol/L bafilomycin or 1 nmol/L concanamycin A. Supernatant and trypsinyzed cells were collected in FACS tubes and centrifuged at 200 g for 5 minutes. The pellet was resuspended in 300 µL of PBS containing 20 µg/mL propidium iodide (PI) and incubated in the dark for 5 minutes. Cells were then analyzed at the FACS (FL2 height) and dead cells were gated by measuring PI positive events. 20000 events were counted.

**Statistical analyses**

The data (mean ± SEM) are representative of 3 independent experiments, performed in triplicates and calculated with Graphpad Prism 5 software.

**Extracellular metabolites analysis.** 300 µL of growth medium of cells incubated as indicated were added to 900 µL of acetonitrile and vortexed for 5 minutes. The suspension was then centrifuged for 10 minutes at 16000 g at 4 °C and the supernatant was submitted to LC-MS analysis.

**Metabolomic analyses**

Intermediates were separated using a liquid chromatography system. The LC system consisted of Finnigan Surveyor Autosampler Plus (ThermoElectron, Hemel Hempstead UK) and MS Pump Plus (ThermoElectron, Hemel Hempstead UK). The column was Sequant Zic-Hilic (150mm X 4.6 mm i.d. 5 microns) with a guard column (20 mm X 2.1 mm i.d. 5 microns) from HiChrom, Reading, UK.

Mobile phase A: 0.1% formic acid v/v in water. Mobile B: 0.1% formic acid v/v in acetonitrile. The flow rate was at 300 μL/minute and gradient was as follow:

0 minutes 80% of B, 12 minutes 50% of B, 26 minutes 50% of B, 28 minutes 20% of B, 36 minutes 20% of B, 37-46 minutes 80% of B.

The autosampler tray temperature was set at 3 °C.

The LC system was coupled online to an LTQ Orbitrap mass spectrometer (ThermoElectron, Hemel Hempstead UK) equipped with an electrospray ionization source set at 4.5 KV in the positive ion and negative ion mode. The capillary was set to 35 V. The heated capillary temperature was set to 250 °C. Tube lense was set to 100 V for positive and -95 V for negative ion modes. The machine was calibrated and tuned with calibration solution freshly before each run. The ion current at every mass to charge ratio (m/z) and elution time was measured by the LC-FTMS. Data acquisition was controlled with Xcalibur 2.0 (ThermoElectron). The mass accuracy was maintained below 1 ppm due to use of a lock mass. The raw chromatograms were then aligned using the software SIEVE™ (ThermoElectron). The integration of the measured ion current over a metabolite’s elution time and m/z interval is directly proportional to its absolute abundance in the solution. We manually removed from the SIEVE’s output unspecific and misaligned peaks to eliminate the noise. In order to identify the peaks, we developed a database mainly from KEGG and METLIN and assigned each metabolite a chemical formula and an identity according to their m/z and retention time (RT) (compared to commercially available standards) by using an Excel macro written in house.

**Statistical analysis of metabolomic results**

For each of two experiments we combined data from the positive and negative settings and used principal components analysis to map the variance in the data. Student’s t-test was used to identify compounds that were significantly different between the hypoxic and normoxic samples. We tested compounds at the 5% level of significance, controlled the false discovery rate (FDR) at 5% and calculated (log) fold changes (FC). Sets of significantly different compounds with the same direction of FC in both experiments were selected for further exploration. Analysis was performed in Partek® Genomics Suite Software, version 6.5 Copyright © 2010 and Bioconductor.
